# Two-Territory Stroke as a Result of Internal Carotid Artery Stenosis and Fetal Posterior Cerebral Artery: A Didactic Paradigm

**DOI:** 10.7759/cureus.50467

**Published:** 2023-12-13

**Authors:** Konstantinos G Seretis, Theofanis Papas, Nikolaos Giannakopoulos, Afroditi Antoniou, Andreas M Lazaris

**Affiliations:** 1 Department of Vascular Surgery, Korgialenio-Benakio Hellenic Red Cross Hospital, Athens, GRC; 2 Department of Vascular Surgery, Attikon University Hospital, Athens, GRC

**Keywords:** internal carotid artery, stroke, fetal type of posterior communicating cerebral arteries, anatomical variations, circle of willis

## Abstract

A complete configuration of the circle of Willis is not always present, and anatomical variations are observed often. The fetal posterior cerebral artery has been described in cases where the embryonic posterior communicating artery has failed to regress, something that may happen on the right side, the left side, or bilaterally. We describe a case of a male patient with bilateral posterior cerebral arteries with direct communication with the internal carotid artery on both sides who presented with symptoms of stroke allocated to both posterior and middle cerebral artery areas. In our knowledge, although there are several reported cases of occipital infarction from internal carotid artery disease, this is the first case of simultaneous infarction in the territories of the posterior cerebral and middle cerebral arteries due to internal carotid artery disease.

## Introduction

The cerebral arterial circle, first described by Willis in 1664, is formed by the afferent arteries in the brain, the left and right internal carotid arteries, and the basilar artery. The circle of Willis is made because the incoming arteries to the brain are interconnected and form the anterior and posterior cerebral arteries. The anterior cerebral arteries are interconnected with the anterior communicating artery, while the basilar artery divides into the two posterior cerebral arteries, which are connected with the internal carotid arteries by the posterior communicating arteries to form the complete circle of Willis. A complete configuration of the circle of Willis is present in about half of the population, and anatomical variations are observed often [1,2]. Fetal posterior cerebral artery occurs in cases where the embryonic posterior cerebral artery has failed to regress and can be found equally on the right and left sides (10% of the population, respectively, for each side) or on both sides (8% of the general population) [3,4].

Atheromatous internal carotid disease is mainly the cause of stroke in the middle cerebral artery distribution area and the borderline area between the middle and anterior cerebral arteries [5]. Occipital infarction is caused mainly by a cardiac source or local embolism from the vertebrobasilar system [6]. On rare occasions, stroke in the distribution area of the posterior cerebral artery can be caused by disease located in the internal carotid artery as a result of the anatomic variation in which the posterior cerebral artery arises directly from the internal carotid artery [7].

We describe a case with both posterior cerebral arteries arising from the internal carotid artery that presented with symptoms from the distribution areas of both the posterior and middle cerebral arteries. In our knowledge, although there are several reported cases of occipital infarction from ICA disease, this is the first case of simultaneous infarction in the territories of the PCA and MCA due to ICA disease.

This article was previously presented as a meeting abstract at the XXVI ESVS 2012 in Bologna, Italy.

## Case presentation

**Figure 1:**
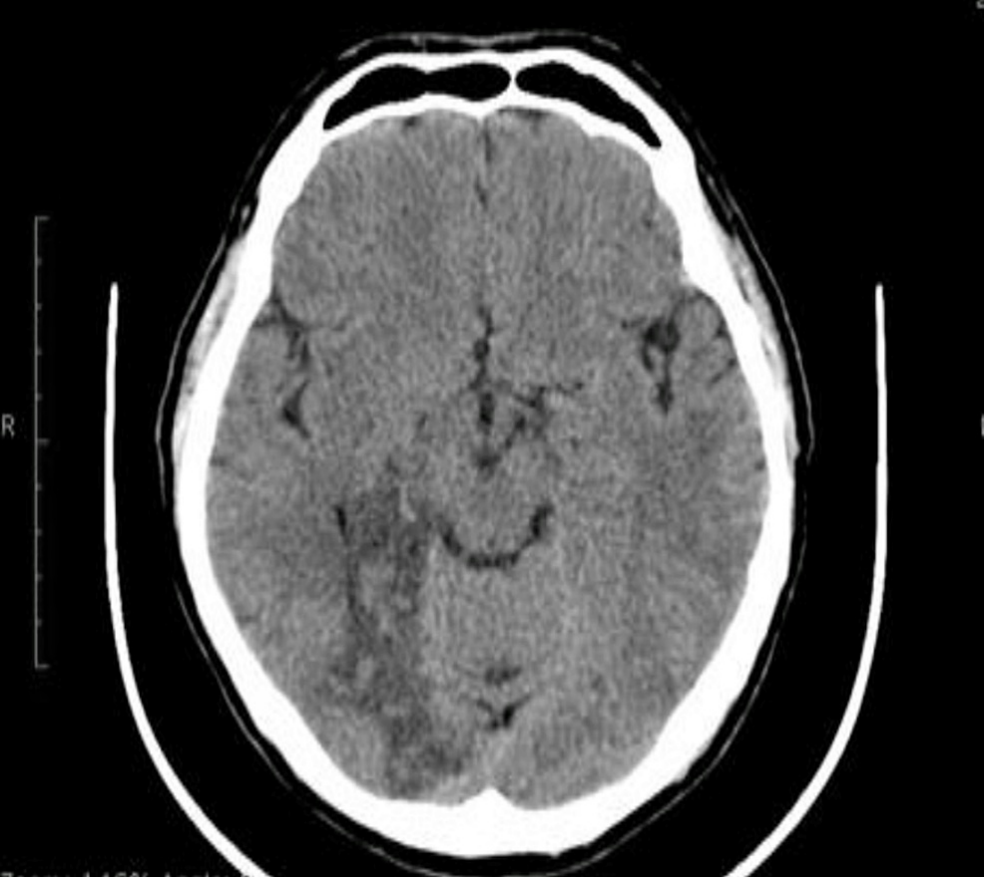
CT scan showing a hypodense region on the right parietal-temporal-occipital region.

A 59-year-old male, a heavy smoker (2-3 packs/day) with a history of hypertension and hypercholesterolemia, was admitted to the emergency department for numbness of the left side of his face and his left hand and leg. He reported an episode of left-side hemiparesis lasting for six hours and a second episode similar to the one that he had on admission, lasting for 12 hours, both episodes in the preceding week before admission. Clinical examination showed left hemianopsia, hypoaesthesia, and slightly decreased muscle power on the left side of his body. The echo study of the heart showed normal left ventricular size and a good systolic function (EF 65%) without any areas of dyskinesia or thrombus, and the Holter study didn’t show any significant arrhythmias. Admission Doppler study of the carotids and the vertebrobasilar circulation showed a near-normal range and flow speed for all vessels examined (RICA PSV 120 cm/sec). An admission CT scan showed a hypodense region on the right parietal-temporal-occipital region, indicating ischemic infarction in the distribution areas of the right posterior and middle cerebral arteries (Figure 1). 

**Figure 2:**
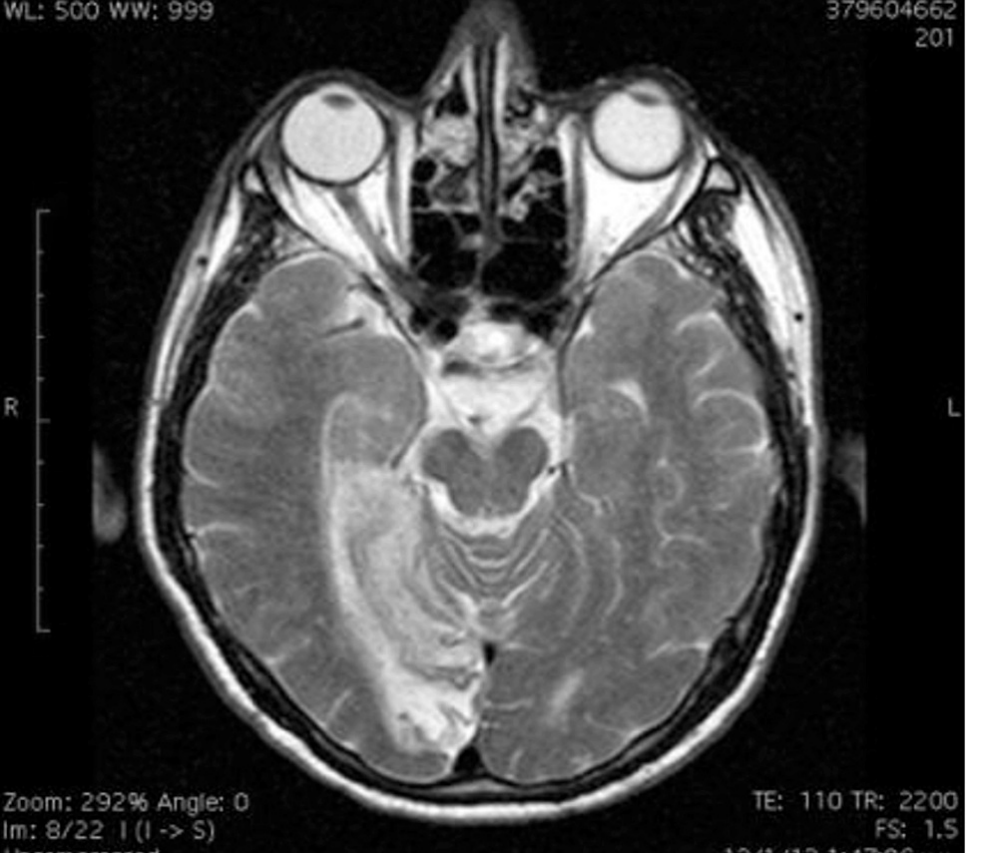
Cerebral MRI showing ischemic infarction in the region of the right occipital lobe, the cognate temporal lobe, the corporis callosi, and the ventral thalamus.

Further evaluation was continued with a cerebral MRI and MRA, with findings of ischemic infarction in the region of the right occipital lobe and, to a smaller extent, in the cognate temporal lobe, the corporis callosi, and the ventral thalamus (Figure 2).

**Figure 3:**
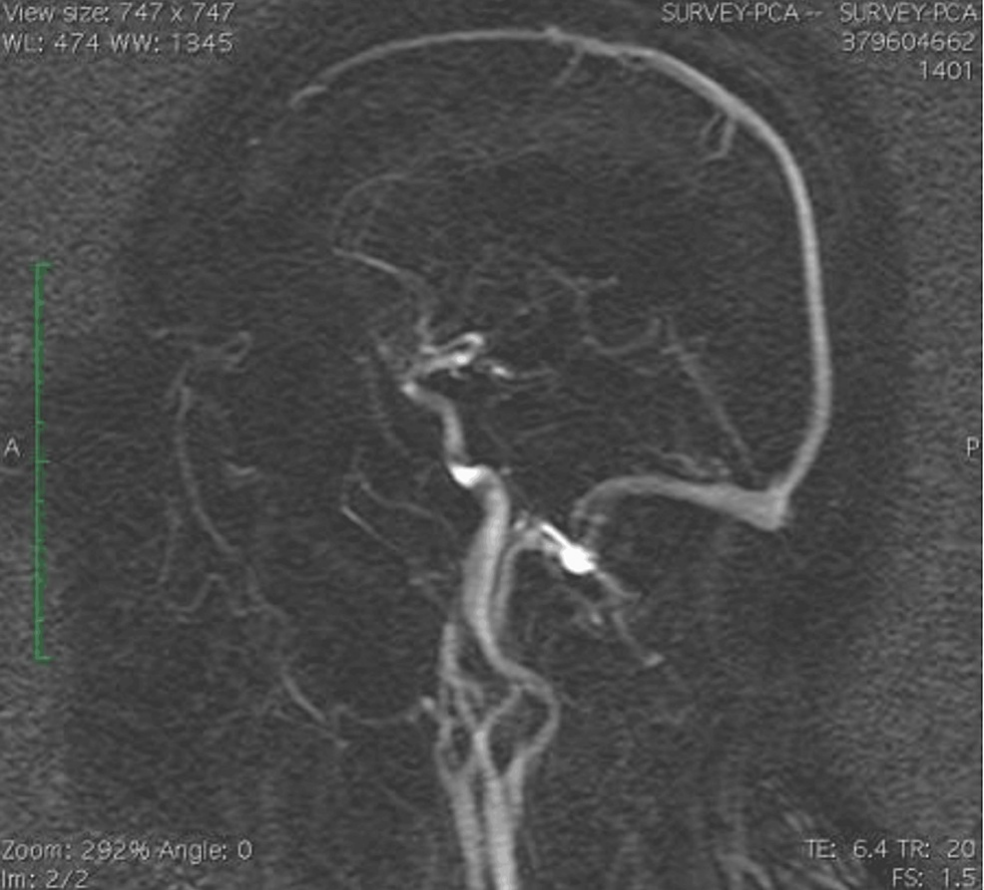
Cerebral MRA reveals the fetal type of both posterior cerebral arteries.

The cerebral MRA revealed the fetal type of both posterior cerebral arteries, arising directly from the internal carotids, without any collateral with the vertebrobasilar system (Figure 3).

**Figure 4:**
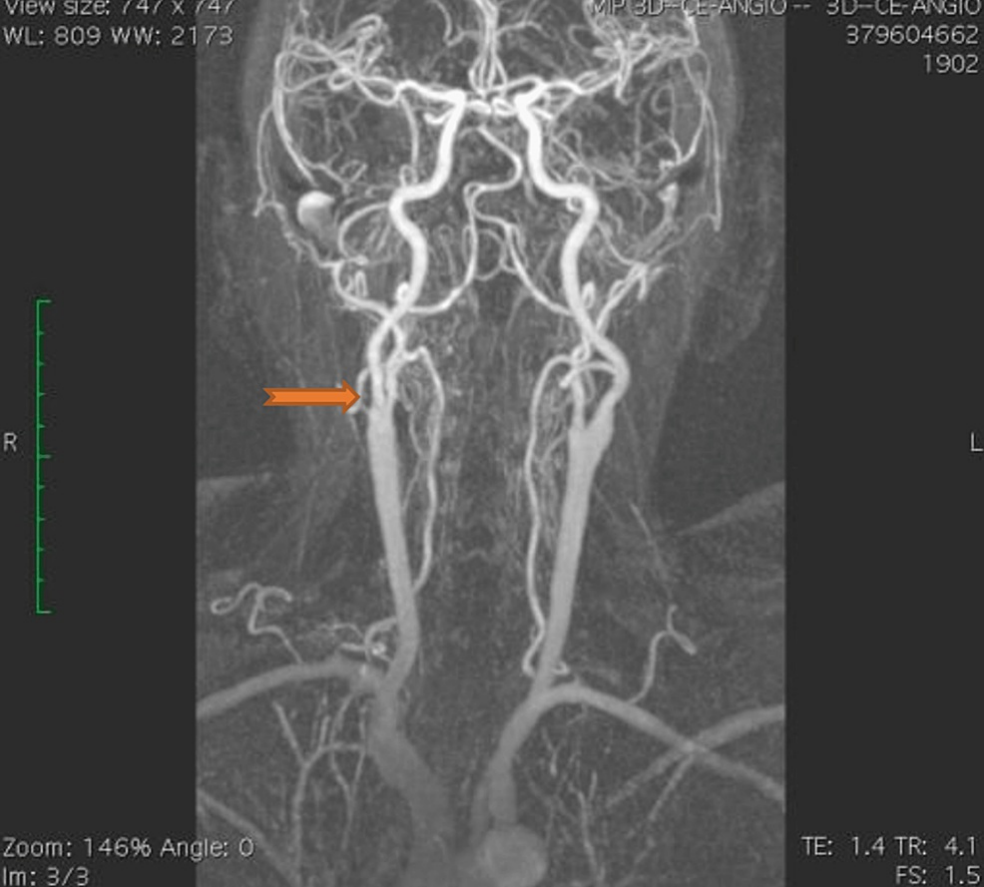
Cerebral MRA, showing a 50%-60% focal stenosis in the region of the right internal carotid bifurcation.

The right internal carotid had an atheromatous plaque, causing 50%-60% focal stenosis in the region of the carotid bifurcation (Figure 4). 

Based on those findings, the patient had a right carotid endarterectomy to remove the potential embolic source. The endarterectomy was performed uneventfully, and the patient was discharged home after three days. 

## Discussion

Occlusive disease of the internal carotid artery in cases of fetal-type posterior cerebral artery may produce occipital infarction. Infarctions in both ICA and PCA territories represent a rare category (1.5% of PCA patients present with hemianopsia). Ischemic lesions in the ICA territory are usually small and multiple, contrary to those found in the PCA territory, which are single and are located mainly in the cortex. Cardioembolic sources are rarely found, and almost all patients demonstrate steno-occlusive lesions of the relevant ICA and fetal-type PCA (fPCA) [8].

Our patient experienced symptoms from the territories of both the posterior and middle cerebral arteries, thus highlighting the anatomic relation between the internal carotid artery and the posterior cerebral artery. The unusual clinical association between the posterior cerebral artery and extracranial internal carotid occlusive disease was present to its full extent in our patient. After the development of the typical signs and symptoms of internal carotid disease, symptoms of occipital infarction followed, originating once again from the internal carotid. The unusual clinical association was only explained when the cerebral MRA revealed fetal type for both posterior cerebral arteries. The fact that both posterior cerebral arteries originated from the internal carotid arteries meant that an embolism from the basilar artery of the posterior circulation was impossible. The only route for passage of an embolus was the anatomic channel of the internal carotid artery to the posterior and middle cerebral arteries. The fetal configuration of both posterior cerebral arteries was also responsible for the development of the occipital infarction because leptomeningeal vessels between the anterior and posterior circulation cannot develop to serve as ‘secondary collaterals’ [9].

The collateral blood flow that is created by the circle of Willis provides a salvage pathway in cases of severe stenosis or occlusion to one of the afferent arteries to the brain. Anatomic variations that produce an absence of collateral blood flow due to hypoplasia or atherosclerosis of the communicating arteries of the anterior and posterior circulation of the brain may lead to a higher risk of stroke. 

## Conclusions

Clinical physicians must be aware of the clinical importance of the collateral function of the circle of Willis and the clinical consequences of anatomic variations that may exist. They should keep in mind that stroke in the territory of the posterior cerebral artery might be associated with severe carotid stenosis, which implies that carotid endarterectomy would be beneficial for that selected group of patients.
